# The ability of *Anopheles gambiae* mosquitoes to bite through a permethrin-treated net and the consequences for their fitness

**DOI:** 10.1038/s41598-019-44679-1

**Published:** 2019-05-31

**Authors:** Gaël Hauser, Kevin Thiévent, Jacob C. Koella

**Affiliations:** Institute of Biology, University of Neuchâtel, Rue Emile-Argand 11, 2000 Neuchâtel, Switzerland

**Keywords:** Ecological epidemiology, Feeding behaviour, Malaria

## Abstract

Insecticide-treated bed-nets (ITNs) control malaria by keeping mosquitoes from reaching people sleeping under a net and by killing mosquitoes. Most tests of ITNs consider their overall epidemiological outcome without considering the different behaviors underlying their effects. Here we consider one of these behaviors: that mosquitoes can bite through the net if its user is touching it. We assayed the ability of an insecticide-sensitive strain of the mosquito Anopheles gambiae to bite through a permethrin-treated or an untreated net, and their subsequent survival and fecundity. Despite the irritancy of permethrin, 71% of the mosquitoes took blood through the ITN (vs. 99% through the untreated net). The ITN reduced the time spent biting, the blood-meal size and the fecundity, and it killed about 15% of the mosquitoes within 24 hours of feeding (vs. 5% on the untreated net). However, the mosquito’s survival was much higher than what we found in WHO cone assays, suggesting that the bloodmeal increased the mosquito’s resistance to the insecticide. Thus, our results suggest that the irritancy and the toxicity of ITNs are reduced when mosquitoes contact and feed on their host, which will affect our understanding of the personal and community protection offered by the ITNs.

## Introduction

Insecticide-treated bed nets (ITNs) are among the most cost-effective tools used to control malaria^1–3^. By reducing the number of malaria cases by 39 to 62% and child mortality by 14 to 29%^1^, they help to save hundreds of thousands of people from dying of malaria every year^4^. The efficacy of ITNs results from several mechanisms of protection.

Bed nets protect people from being infected by malaria by creating a physical barrier between the user and mosquitoes. Mosquitoes can bite the user only if they find a hole through which they can penetrate the net or if they find a patch of skin that is touching the net and that they can bite through the net. Treating the net with an insecticide has several additional effects. First, ITNs can repel mosquitoes, so that they are less likely to approach the user. Second, they irritate mosquitoes, so that upon contact with an ITN some mosquitoes fly away rather than moving along the net to find a hole^5–10^. Third, if mosquitoes touch the net long enough (when they are trying to bite or when then they are resting on it after having bitten), the insecticide may kill them. By decreasing the number of infectious mosquitoes, this insecticidal effect offers community-wide protection^11,12^ in addition to the personal protection.

The relative importance of the mechanisms of protection depends on the insecticide. Permethrin, for example, is only slightly spatially repellent but strongly irritant^13^. In one study in Tanzania, for example, treating a bed net with permethrin had almost no effect on the number of mosquitoes that entered experimental houses, but reduced the probability that a mosquito passed through a net by a factor of about 8^10^. Nevertheless, the insecticide reduced blood-feeding success only by a factor or about 3^10^, which suggests that many mosquitoes bit through the ITNs without penetrating them. Indeed, although permethrin may keep mosquitoes from biting through the net, a laboratory experiment (with few mosquitoes) suggests that complete protection requires a higher concentration than what is used in commercially available ITNs like Olyset (1 g/m^2^)^14^; at 0.8 g/m^2^ (slightly more than what was found on an Olyset net after 1 year of use^15^) more than half of the mosquitoes were able to bite through the net.

That mosquitoes can bite through insecticide-impregnated nets despite being irritated weakens the personal protection offered by the irritancy. Community protection may, however, be maintained if mosquitoes die after they have bitten through an ITN, preventing further infectious bites. In the study mentioned above, about a third of the mosquitoes that managed to bite through the net were knocked down and died^14^. This short-term effect of exposure to insecticides during biting may be complemented by long-term or sub-lethal effects. Thus, exposure to a Permanet 2.0 (a net treated with 0.5% deltamethrin) reduces the survival rate of mosquitoes for several days after exposure^16^. Mosquitoes irritated by insecticides are likely to stop their blood-seeking behavior for several days^17^ (thus protecting the ITN-users and others from being bitten) and lay fewer eggs^17^ (potentially reducing the number of mosquitoes in the population).

The aim of this study was to bring these ideas together and extend them by evaluating the ability of insecticide-sensitive *A*. *gambiae* mosquitoes to bite through a new generation of ITN and its consequences for several aspects of the mosquitoes’ feeding behavior and fitness. While the efficacy of ITNs to reduce malaria prevalence is not questioned here, our goal was to understand better the properties and effects of an ITN in the scenario of mosquitoes having access to a human host by biting through the net. Also, using a sensitive strain of *A*. *gambiae* allowed us to establish the maximal level of protection the ITN can offer in this particular case. As a model, we used an ITN that is widely used in Africa: Olyset Plus®. This ITN is treated with 2% (w/w) permethrin and 1% piperonyl butoxide (PBO), which slows down the metabolic degradation of pyrethroids^18,19^. The presence of PBO allows this ITN to remain efficient against mosquitoes harboring metabolic resistance^9^, which is particularly important in malaria endemic regions, where resistance is now wide-spread^20^. We also expect that the slow degradation of the insecticide might increase the effects of the insecticide on the mosquito’s survival several days after exposure.

Here we measured the proportion of mosquitoes that were able to take blood through an untreated net and through an Olyset Plus net, the time mosquitoes spent blood-feeding on the nets, and their blood-meal size. We also measured the mortality of the mosquitoes within 24 hours of their blood-meal and the fecundity and longevity of the surviving mosquitoes. In a separate experiment we measured the resistance level of unfed and freshly blood-fed mosquitoes exposed to the Olyset Plus net, so that we could test whether the act of blood-feeding affected the survival of the mosquitoes contacting the ITN.

## Methods

### Mosquitoes

We used the insecticide-sensitive Kisumu strain of *Anopheles gambiae s*.*s*. originating from western Kenya^21^. We confirmed the sensitivity of our colony by exposing 100 mosquitoes to 0.75% permethrin (WHO filter papers)^22^ for one hour and finding that all mosquitoes died within 24 hours. Throughout our experiment, the mosquitoes were kept in an insectary maintained at 26.5 ± 0.5 C° and 70 ± 5% humidity with a 12:12 hours dark:light cycle.

### Effect of ITN on blood-feeding behavior

We selected mosquito larvae haphazardly the day they hatched and reared them individually in 12-well-plates with the standard food regime of our lab: day of hatching, 0.04 mg TetraMin Baby® fish food per larvae; 1 day after hatching, 0.06 mg; day 2, 0.08 mg; day 3, 0.16 mg; day 4, 0.32 mg; day 5 and more, 0.6 mg. Pupae were moved to 21 × 21 × 21 cm plastic cages and adults were provided with a 6% sucrose solution.

Three to four days after emergence, we moved mosquitoes individually to 120 mL plastic cups covered with either a permethrin-treated net (Olyset Plus®) or an untreated net (Pharmavoyage® Trek) and gave them the opportunity to blood-feed for 8 min on Gaël Hauser's (GH) arm. We measured the duration of their blood-meal as the difference between the time at which they started to probe and the time they pulled their stylet from the arm. Directly after the blood meal, we assessed the blood-meal size visually, removed unfed mosquitoes, moved blood fed mosquitoes to individual 30 ml plastic tubes covered with an untreated net and let them have access to a cotton ball soaked in a 6% solution of sucrose. Twenty-four hours after the blood meal, we recorded the number of dead mosquitoes. Three days after the blood meal, we moved the mosquitoes to 120 mL individual cups that contained wet filter paper, and collected the eggs laid onto the filter paper the next day. To quantify hematin, we diluted the faeces that had been excreted in the 30 mL tubes in 1 mL 1% lithium carbonate (method described below). Every day, we assessed the survival of the mosquitoes.

#### Blood meal size

As previously described by Briegel and coworkers^23^, we added 1 mL of 1% solution of lithium carbonate on the faeces contained in the 30 mL tubes and gently mixed the solution with a pipet until complete elution. The solutions were then transferred to 1.5 mL eppendorf tubes and kept at 4 °C until assayed. We then vortexed the eppendorf tubes and transferred 200 µL of each solution to an ELISA plate along with a serial of standard dilutions of known haematin concentration, and we measured the absorbance at 387 nm with an ELISA plate reader. Each sample was measured in duplicate on 2 different plates. We calculated the haematin concentration from the standard curve specific to each plate and used the average concentration of the two replicates of each sample for the statistical analyses. Standards dilutions used porcine haematin (Sigma-Aldrich®, Saint-Louis, Missouri) with 8 different concentrations ranging from 0 to 50 ug of hematin per mL. Repeatability was 0.98 (calculated from replicated samples).

#### Body size

Wing length was used as a proxy for the mosquito’s body size. We placed the wings onto a slide, took a digital photograph of each wing and measured it with the software ImageJ from the distal end of the alula to the tip of the wing (the end of the vein R3) without the fringes.

### Effect of bloodmeal on resistance

We reared larvae in groups of 200 in 35 × 15 × 5 cm trays containing 800 ml deionized water. This density limits competition among larvae^24^. We fed them with the standard food regime of our lab (described above). We moved pupae to 21 × 21 × 21 cm plastic cages and provided adults with a 6% sucrose solution. Four days after the first mosquitoes emerged, we blood-fed approximately 250 females for 8 minutes on GH’s arm. Directly after the blood meal, we measured the resistance of these mosquitoes and of approximatively 250 unfed females with the WHO cone bioassay^25^. We placed the mosquitoes in groups of 5 into a plastic cone (the upper 15 cm of a PET bottle of 8 cm diameter) fixed on a piece of an Olyset Plus bednet. We exposed the mosquitoes for 1.5, 3, 5, 8, or 12 min and recorded mortality 24 h after exposure. We replicated the exposure of 5 mosquitoes 10 times for each duration of exposure. To control for mortality induced by the manipulation itself, we also tested 10 replicates of 5 fed mosquitoes and 10 replicates of 5 unfed mosquitoes on an untreated net during 12 min. We did not find any dead mosquito after 24 h in these control replicates.

### Statistical analysis

For ANOVAs and LMs described below, the normality of model residuals was visually assessed and heteroskedasticity was checked with the *bptest* function (from *lmtest* library in R^26^). For Cox models, the assumption of proportional hazards was tested with the *cox*.*zph* function from the *survival* library. All analyses and graphs were done with the software R (version 3.4.4)^27^ and with the Rstudio interface^28^ (version 1.1.456). Graphs were made using R, and edited (labels, colors and format) using Inkscape (version 0.92.2).

### Effect of ITN on blood-feeding behavior

All data collected during the feeding assay and following measurements, including haematin concentration, eggs number, survival and wing length can be found as Supplementary Data S1.

#### Blood-meal size

We described the blood-meal in five ways: (1) the proportion of mosquitoes that tried to bite at least once (*biting success*), (2) the proportion that succeeded to take blood during the 8 minutes they were allowed to (*feeding success*), (3) the time required for the mosquitoes to start biting through the net (*time to bite*), (4) the time they spent feeding until they detached or until the end of the allocated time (*feeding time*), and (5) the quantity of haematin in the faeces, used as a proxy for the quantity of blood ingested (*haematin level*).

Biting and feeding success (binomial response variable) were analyzed with a Generalized Linear Model (GLM) with binomial error distribution. Time to bite was analyzed with a Cox proportional hazard regression model (from the package *survival* in R^29^), where the mosquitoes that did not try to bite were censored. Feeding time was analyzed with a Cox proportional hazards regression model, where the mosquitoes that were still feeding at the end of the allocated eight minutes were censored. Haematin level was analyzed with an ANOVA. Each model included the type of net (untreated or Olyset Plus) as an explanatory factor.

#### Fecundity and longevity

Fecundity was analyzed as: (1) the proportion of mosquitoes that laid at least one egg (*laying success*), and (2) the number of eggs laid by each female.

Laying success was analyzed with a GLM with binomial error distribution, where the type of net was included as explanatory factor and wing length was included as covariable. The number of eggs was analyzed with a multiple regression that included the type of net, wing length and haematin level as explanatory variables. Non-significant interactions were dropped from the final model.

Longevity was tested in two different ways: (a) the proportion of mosquitoes that survived the 24 h following blood feeding (that is, the standard way of assessing the effect of insecticides on mortality), and (b) longevity of mosquitoes that survived the first 24 hours (that is a delayed effect of the insecticide). Survival after 24 h was analyzed with a GLM with binomial error distribution. Longevity was analyzed with a Cox proportional hazards regression model. Both analyses included the type of net as an explanatory factor.

### Effect of bloodmeal on resistance

We built time-response models with the *drm* function (*drc* library in R^30^). We used a 2 parameter log-logistic function, setting higher and lower limits for time-mortality curves to 1 and 0. Statistical comparison of the values of LT90 of each treatment was done with the *EDcomp* function of the *drc* package. All data collected to compute the time-response models can be found as Supplementary Data S2.

## Results

### Effect of ITN on blood-feeding behavior

#### Blood meal

101 mosquitoes were tested on an untreated bed net and 85 on an Olyset Plus net. All of the 101 mosquitoes tested on an untreated net tried to bite (100% (95% CI: 95 to 100%)), whereas on the Olyset Plus net, 75 out of the 85 tried (88% (95% CI: 79 to 94%)) (X^2^ = 16.34, df = 1, p < 0.001). Moreover, 100 out of the 101 mosquitoes tested on the untreated net obtained some blood (99% (95% CI: 95 to 100%)), whereas only 60 out of 85 obtained a blood-meal through the Olyset Plus net (71% (95% CI: 60 to 80%)) (X^2^ = 34.42, df = 1, p < 0.001). If mosquitoes tried to bite, it took them an average of 35.0 seconds (±12.9 (95% CI)) to start biting through the untreated net, but about 50% 53.5 seconds (±13.6) to start biting through the Olyset Plus net (X^2^ = 25.4, df = 1, p < 0.001).

**Figure 1:**
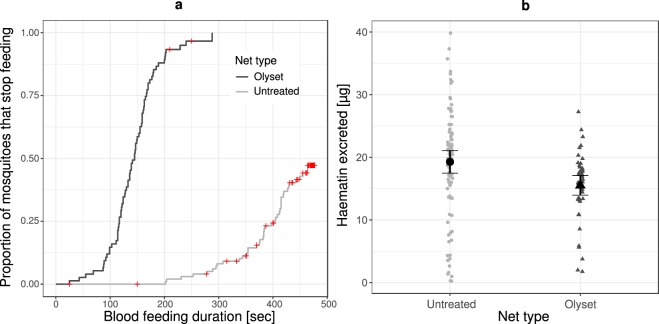
Blood feeding duration and subsequent haematin excretion. (**a**) Cumulative proportion of mosquitoes with a given duration of blood-feeding (time between the beginning of probing and detaching). Red crosses represent the mosquitoes that did not spontaneously detach and were thus still biting at the end of the allocated time. (Untreated: N = 101, Olyset: N = 75). (**b**) Quantity of haematin excreted by mosquitoes in function of the type of net they were able to feed through (Untreated: N = 92, Olyset: N = 46). Error bars show the 95% confidence intervals.

More than 75% of the mosquitoes that bit through an untreated net were still feeding 400 sec after they had started to bite, whereas about 75% of the mosquitoes biting through an Olyset Plus net stopped feeding after 175 sec and none of them fed for more than 300 seconds (Fig. 1a; X^2^ = 198.35, df = 1, p < 0.001).

Feeding time was positively correlated to the amount of haematin excreted by the mosquitoes after blood digestion (F_1,136_ = 652.2, p = 0.001). Consequently, the amount of excreted haematin was 23% higher for mosquitoes that had fed through the untreated net (19.1 µg ± 1.9 (95% CI)) than for those that had bitten through the Olyset Plus net (15.5 µg ± 1.6) (Fig. 1b; F_1,136_ = 6.8, p = 0.01).

#### Fecundity and survival

**Figure 2:**
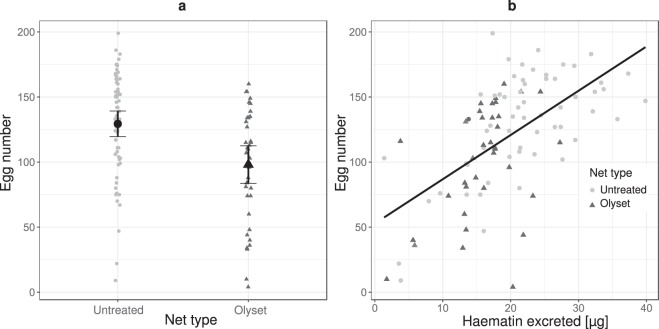
Fecundity and its correlation with haematin level. (**a**) Quantity of eggs laid by mosquitoes in function of the type of net they bit through (Untreated: N = 63, Olyset: N = 37). Error bars show the 95% confidence intervals. (**b**) Relationship between the number of eggs laid by mosquitoes and the quantity of haematin they excreted. Black triangles represent the mosquitoes that fed through the Olyset plus net, and the grey dots represent those that fed through the untreated net (Untreated: N = 59, Olyset: N = 36). The line shows the linear regression.

The proportion of mosquitoes that laid eggs was 68% (95% CI: 58.4 to 77.1%) if they had fed through an untreated net and 77% (95% CI: 63.4 to 86.7%) if they had fed through an Olyset Plus net (X^2^ = 1.7, df = 1, p = 0.19). Egg-laying success tended to increase with the mosquitoes’ wing length (X^2^ = 3.79, df = 1, p = 0.051). In contrast, among the females that laid eggs, the number of eggs laid per female (was greater if the mosquitoes had bit through an untreated net (129 ± 10 (95%CI)) than if they had bitten through Olyset Plus (98 ± 14) (Fig. 2a; F_1,96_ = 13.14, p < 0.001). The number of eggs increased with the mosquito’s wing length (F_1,96_ = 9.78, p = 0.002).

In a second model, haematin level was included in the multiple regression to look at possible mechanisms responsible for the observed difference. Haematin excretion level was positively correlated with eggs number (Fig. 2b; F_1,91_ = 36.56, p < 0.001), and that the type of net no longer had a statistically significant effect (F_1,91_ = 2.44, p = 0.12). The interaction between net type and haematin level was not significant (F_1,90_ = 0.62, p = 0.43) and was therefore removed from the model.

**Figure 3:**
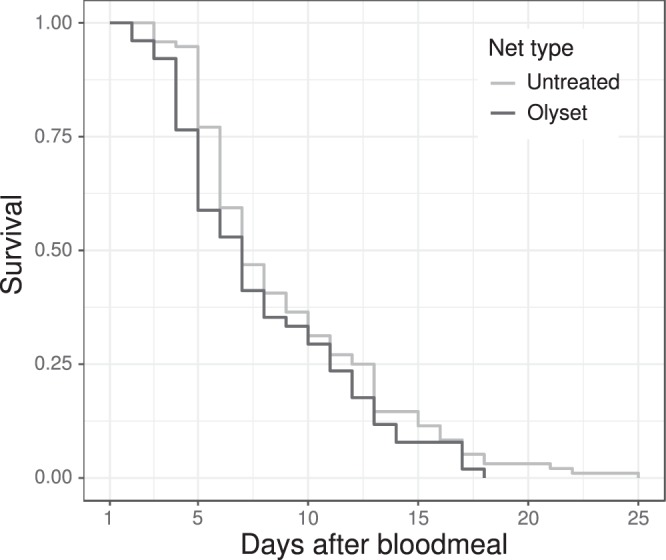
Mosquito’s longevity in function of the type of net they could feed through. Survival is shown starting from 24 h following the blood meal (day 1). The black line represents the survival of mosquitoes that bit through the Olyset net; the grey one represents the mosquitoes that fed through the untreated net (Untreated: N = 96, Olyset: N = 51).

96 out of the 100 (96%) mosquitoes that had fed through an untreated net survived the 24 h after their blood meal, whereas only 51 out of 60 (85%) of the mosquitoes that had fed through an Olyset Plus did (X^2^ = 5.87, df = 1, p = 0.015). Once they had survived the first 24 h, half of the mosquitoes died within 6 days, whether they had fed through the ITN or through the untreated net (Fig. 3; X^2^ = 1.79, df = 1, p = 0.18).

### Effect of bloodmeal on resistance

**Figure 4:**
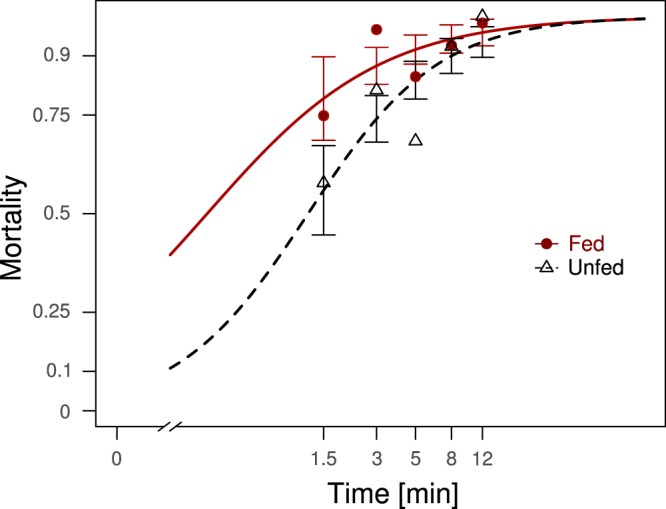
Time–mortality curve of unfed (black dashed line) and freshly blood fed (red solid line) females exposed to an Olyset Plus net following the WHO cone bioassay procedure. Points represent averages mortality at each time, five exposure durations were assessed for both unfed and fed mosquitoes. Error bars show the 95% confidence intervals of the regression lines.

251 fed and 287 unfed females were tested on an Olyset Plus net. The LT90 (duration of exposure that killed 90% of the mosquitoes within 24 hours) was estimated as 8′01″ for unfed females and 4′01″ for fed ones (t = −3.01, p = 0.002, Fig. 4).

## Discussion

Insecticide treated bed-nets prevent mosquitoes from biting their user by irritating them. With this study, we confirm the permethrin of the Olyset Plus net to be irritant for mosquitoes^5,13,31^. However, despite that irritancy, 88% of the mosquitoes tried to bite and 71% succeeded to take a blood meal through the net. This feeding through the permethrin-treated net came with fitness costs for the mosquitoes, for it reduced their chance to survive and their fecundity, corroborating the results from previous studies^14,16,17^.

The irritancy of the Olyset plus net was confirmed by the fact that the mosquitoes took more time to start blood feeding and spent less time feeding through the treated net than through the untreated net. Although we could not distinguish the role of spatial repellency and contact irritancy for the delay in the time mosquitoes took to bite, the reduced time mosquitoes spent biting through the net confirms that permethrin is strongly irritant^5,13,31^. In addition, by reducing the duration of the blood meal, the irritancy of the Olyset Plus net decreased the quantity of blood mosquitoes were able to take and, as a consequence, the number of eggs they laid. We thus also confirmed that sub-lethal exposure to pyrethroids may alter the number of eggs mosquitoes are able to produce^17^. Our results, however, suggest that this reduction of fecundity after an ITN exposure is not a direct effect of the toxicity of the insecticide, but rather caused by a reduced feeding time. Overall, our results suggest that the ITN may not only prevent mosquitoes from biting by irritating them, but may also reduce the density of mosquitoes through a reduction of their fecundity.

Although the Olyset Plus net strongly irritated the mosquitoes, it prevented only 12% of the mosquitoes from trying to bite through the net, and 80% of the ones that tried to bite were able to take blood. Therefore, when the user of net touches the net, the personal protection offered by it may be reduced, which would have strong implications for the epidemiology of mosquito-borne diseases like malaria, if our results were confirmed in the field. Indeed, since *Plasmodium* sporozoites (the stage that is infectious for human) are mostly released at the beginning of the bite, the large proportion of mosquitoes that bite, even if only for a short time, will keep the the transmission potential high^32,33^. It is, however, worth noting that our study focused on the ability of mosquitoes to bite through an Olyset Plus net, and did not consider other factors that may reduce the number of mosquitoes reaching the net in field condition, like long range repellency.

Since Olyset Plus is treated with 1% piperonyl butoxide (PBO), which slows down the metabolic degradation of pyrethroids^18,19^, we expected to see an effect of exposure to the insecticide on survival throughout the mosquito’s life. However, long-term mortality was not affected by the ITN, and that there was only a difference with the untreated net at 24 h after the blood meal. Thus, despite PBO, permethrin appeared to have only a short-term impact on survival.

Because the WHO recommend that ITNs kill 80% of the mosquitoes within 24 h after being exposed for a duration of 3 min in a cone assay^25^, we expected that most of the mosquitoes would not survive after a blood meal through the Olyset Plus net. However, we found a surprisingly low impact on survival: only 15% mortality at 24 h. Indeed, according to our dose-response model, mosquitoes being exposed to an Olyset Plus net during 2 min 23 s – the average feeding time recorded in the blood feeding experiment– should have experienced 68% (unfed females) or 85% (fed females) mortality at 24 h. A possible explanation for the high survival is that, contrarily to the mosquitoes used in the cone bioassay, which were all exposed in a similar way, the mosquitoes used in the feeding experiment had the choice to bite (and being exposed) and bit for a variable amount of time. Thus, it might be that only the most resistant individuals succeeded to take a bloodmeal, which may explain why they also survived to the contact with the net. However, even if we assume that all the mosquitoes that did not bite would have bitten and then died within 24 h, the recorded mortality would only have been increased from 15% to 39%, which is still considerably lower than what was predicted by the cone assay for a comparable time of exposure.

Another possibility to explain the low mortality induced by the blood meal through the treated net may come from the blood meal itself. Particularly, we hypothesize that when both the blood meal and permethrin exposure happen simultaneously, it helps mosquitoes to reduce the detrimental effect of permethrin. Indeed, the presence of blood alone in the midgut cannot explain the low mortality experienced by the individuals that blood fed through the Olyset Plus net, for the fed mosquitoes were found to be more sensitive than unfed mosquitoes in the cone assay. Therefore, we briefly introduce two possible mechanisms that might be implied. First, both a blood meal^34^ and pyrethroid exposure^35,36^ increase the concentration of reactive oxygen species (ROS), which is quickly followed by a higher expression of different antioxidants in the midgut and the fat body^34^, as found in some pyrethroid-resistant mosquito strains^37^. Thus, one possibility may involve an oxidative based interplay between the blood meal and permethrin exposure, which may reduce the damage induced by the insecticide. However, the fact that this mechanism would no longer be active a few minutes after the blood meal, as we tested in the cone assay, would be puzzling. A second possibility may involve temperature. During a blood meal the body temperature of mosquitoes increases rapidly^38^. Because the toxicity of pyrethroids decreases at higher temperatures^39–41^, blood-feeding mosquitoes might suffer less from being exposed to pyrethroids. This hypothesis is also more consistent with the results obtained via the cone bioassay: fed mosquitoes had already cooled down when they were put on the Olyset Plus net and therefore were no longer protected by the high temperature reached during the blood meal.

The possibility for the blood meal to mitigate toxicity of pyrethroids when mosquitoes are biting through the net may be of high importance when evaluating ITNs efficiency and their insecticidal properties. Indeed, our results suggest that the protection they offer might be impeded in that particular case. Although we confirmed that the commercially available permethrin-treated Olyset Plus net was irritant, 88% of the tested mosquitoes tried to bite through the net and most of them succeeded to take blood and further survive. It follows that mosquitoes that have the possibility to bite through an Olyset Plus net may potentially acquire one or more parasites and transmit them, affecting both the personal and community protection that the net confers in standard experimental conditions^9^.

To conclude, while the efficiency of Olyset Plus net to reduce malaria prevalence has already been demonstrated, our results showed that the insecticide itself only slightly prevents mosquitoes from biting through the net when they are given the opportunity. The time mosquitoes spent feeding on the net was reduced due to its irritant property, which reduced mosquito fecundity. Finally, taking a blood meal helped mosquitoes to survive their exposure to permethrin through a physiological mechanism that remains to be determined. Altogether, our results point out the importance to avoid skin contact with the net to guarantee a maximal protection for both the user and the community.

## Supplementary information

Dataset S1

Dataset S2

## Data Availability

All data generated or analysed during this study are included as Supplementary Information files.
